# Understanding author choices in the current conservation publishing landscape

**DOI:** 10.1111/cobi.14369

**Published:** 2024-09-03

**Authors:** Natalie Yoh, Mukhlish Jamal Musa Holle, Jasmin Willis, Lauren F. Rudd, Iain M. Fraser, Diogo Veríssimo

**Affiliations:** ^1^ Durrell Institute of Conservation and Ecology University of Kent Canterbury UK; ^2^ Nelson Institute for Environmental Studies University of Wisconsin–Madison Madison Wisconsin USA; ^3^ Department of Biology University of Oxford Oxford UK; ^4^ Faculty of Biology Gadjah Mada University Yogyakarta Indonesia; ^5^ School of Economics University of Kent Canterbury UK

**Keywords:** academic societies, article processing charges, discrete choice experiment, double‐blind review, impact factor, open‐access, peer review, publishing preferences, acceso abierto, experimento de elección discreta, factor de impacto, preferencias editoriales, revisión doble ciego, revisión por pares, sociedades académicas, tasas de tramitación de artículos, 野生动物共存, 动物群内互动, 顶级捕食者, 资源分配, 人类与野生动物冲突, 结构方程建模, 新型生态系统

## Abstract

Conservation literature addresses a broad spectrum of interdisciplinary questions and benefits. Conservation science benefits most when a diverse range of authors are represented, particularly those from countries where much conservation work is focused. In other disciplines, it is well known that barriers and biases exist in the academic publishing sphere, which can affect research dissemination and an author's career development. We used a discrete choice experiment to determine how 7 journal attributes affect authors’ choices of where to publish in conservation. We targeted authors directly by contacting authors published in 18 target journals and indirectly via communication channels for conservation organizations. We only included respondents who had previously published in a conservation‐related journal. We used a multinomial logit model and a latent class model to investigate preferences for all respondents and distinct subpopulations. We identified 3 demographic groups across 1038 respondents (older authors from predominantly middle‐income countries, younger authors from predominantly middle‐income countries, and younger authors from high‐income countries) who had published in conservation journals. Each group exhibited different publishing preferences. Only 2 attributes showed a consistent response across groups: cost to publish negatively affected journal choice, including authors in high‐income countries, and authors had a consistent preference for double‐blind review. Authors from middle‐income countries were willing to pay more for society‐owned journals, unlike authors from high‐income countries. Journals with a broad geographical scope that were open access and that had relatively high impact factors were preferred by 2 of the 3 demographic groups. However, journal scope and open access were more important in dictating journal choice than impact factor. Overall, different demographics had different preferences for journals and were limited in their selection based on attributes such as open access policy. However, the scarcity of respondents from low‐income countries (2% of respondents) highlights the pervasive barriers to representation in conservation research. We recommend journals offer double‐blind review, reduce or remove open access fees, investigate options for free editorial support, and better acknowledge the value of local‐scale single‐species studies. Academic societies in particular must reflect on how their journals support conservation and conservation professionals.

## Abstract (es)

Comprensión de las elecciones de los autores en el entorno actual de publicaciones sobre la conservación

**Resumen:**

La bibliografía sobre conservación aborda un amplio espectro de preguntas y beneficios interdisciplinarios. La mayor parte de ella representa una gama diversa de autores, sobre todo de países en los que se centra gran parte del trabajo de conservación. Es bien sabido que en otras disciplinas existen barreras y sesgos en el ámbito de la publicación académica que pueden afectar a la difusión de la investigación y al desarrollo de la carrera de un autor. Usamos un experimento de elección discreta para determinar cómo afectan siete atributos de las revistas sobre conservación en la elección de los autores sobre en cuál publicar. Nos dirigimos directamente a los autores y nos pusimos en contacto con quienes publicaban en 18 revistas objetivo e indirectamente a través de los canales de comunicación de las organizaciones de conservación. Sólo incluimos a los encuestados que habían publicado anteriormente en una revista relacionada con la conservación. Usamos un modelo logit multinominal y un modelo de clases latentes para investigar las preferencias de todos los encuestados y de las distintas subpoblaciones. Identificamos tres grupos demográficos entre los 1038 encuestados (autores de más edad de países con predominancia de ingresos medios, autores más jóvenes de países con predominancia de ingresos medios y autores más jóvenes de países con ingresos altos) que habían publicado en revistas de conservación. Cada grupo mostraba preferencias editoriales diferentes. Sólo dos atributos mostraron una respuesta coherente en todos los grupos: el costo de la publicación afectaba negativamente a la elección de la revista, incluidos los autores de países con ingresos altos, y los autores tenían una preferencia coherente por la revisión doble ciego. Los autores de países con ingresos medios están dispuestos a pagar más por las revistas pertenecientes a la sociedad, a diferencia de los autores de países de ingresos altos. Dos de los tres grupos demográficos prefieren las revistas de ámbito geográfico amplio, de acceso abierto y con un factor de impacto relativamente alto. Sin embargo, el alcance de la revista y el acceso abierto fueron más importantes que el factor de impacto. En general, los distintos grupos demográficos tenían preferencias diferentes en cuanto a las revistas y su selección se veía limitada por atributos como la política de acceso abierto. No obstante, la falta de encuestados procedentes de países con bajos ingresos (2% de los encuestados) destaca las barreras generalizadas para la representación en la investigación sobre conservación. Recomendamos que las revistas ofrezcan revisiones doble ciego, reduzcan o eliminen las tarifas de acceso abierto, investiguen opciones de apoyo editorial gratuito y reconozcan mejor el valor de los estudios de una sola especie a escala local. Las sociedades académicas, en particular, deben reflexionar sobre la forma en que sus revistas apoyan la conservación y a los profesionales de la conservación.

## 对人类干扰的不同容忍度对顶级捕食者优势相互作用的影响

**【摘要】** 人类活动可能会改变猫科动物的群落结构, 促进对人类活动容忍度高的物种(通常是中型捕食者)续存, 排斥生态需求高的物种(顶级捕食者), 从而改变物种共存规则。本研究旨在确定人类活动如何影响顶级捕食者之间的群内关系及其对中型捕食者的级联影响。尽管已有证据表明顶级捕食者在减少, 但人们对这些影响仍知之甚少。我们在大陆尺度使用结构方程模型研究了栖息地质量和数量、家畜密度, 以及其他人类压力如何改变新热带地区处于食物链顶端的三个物种——美洲虎(*Panthera onca*)、美洲狮(*Puma concolor*)和豹猫(*Leopardus pardalis*)的群内关系。我们将来自系统研究的猫科动物存在‐不存在数据以0.0833º的分辨率整合到了*Neocarnivores*数据集中。研究发现, 人类干扰导致美洲虎出现概率降低了‐0.35个标准差。出乎意料的是, 绵羊(*Ovis aries*)或山羊(*Capra aegargrus hircus*)和美洲虎的出现概率与美洲狮出现概率呈正相关, 而美洲狮的出现则与豹猫的出现呈负相关。森林覆盖面积对美洲虎(β = 0.23)和豹猫(β = 0.12)的出现影响大于保护地面积, 而保护地面积没有显著影响。人类活动对美洲狮的出现没有影响, 而小型家畜的出现则有积极影响, 这支持美洲狮比美洲虎更能适应栖息地干扰的观点。我们的研究结果表明, 人类干扰有可能逆转大型猫科动物之间的等级竞争优势, 导致群落结构不平衡。这种变化使美洲虎处于不利地位, 并提高了美洲狮在群落等级中的地位, 还导致豹猫被排除在外, 尽管其对人为干扰的敏感性相对较低。以上结果表明, 保护工作应该从保护地扩展到周边景观, 因为这些地区的复杂性和潜在冲突更加明显。**【翻译:胡怡思;审校:聂永刚】**

## INTRODUCTION

Academic publishing is considered central to the dissemination of scientific research (Medina‐Franco & López‐López, 2022). Academic publications provide a foundation of scientific understanding to inform on‐the‐ground conservation strategies (Stirling & Burgman, 2021). As well as research dissemination, publishing can also be important for a researcher's career progression. The perceived quality of academic journal publications can affect a researcher's likelihood of accessing future funding, promotions, and their perceived legitimacy as a researcher (Hall & Page, 2015). For researchers employed by organizations, such as nonprofit organizations, publishing in reputable journals also increases the visibility of the organization and can be used to document impact. Therefore, authors must consider how journal choice will ensure the dissemination of their findings and how it will contribute to their careers and potentially benefit their organization.

Researchers face multiple considerations and challenges when choosing where to publish, including navigating the many barriers and biases that exist within the publishing sphere. From the author's perspective, such challenges can be divided into internal and external barriers. Internal barriers may include pressure to conform to Westernized journal styles (Hazen, 2016; Oshiro et al., 2020; Prasojo et al., 2019), whereas external barriers may include bias against authors (e.g., geographical or gender discrimination) during the review process and biases in the perceived value of the research (e.g., journal scope) (Smith et al., 2023; Tomkins et al., 2017). For example, the conservation literature is still considerably biased toward authors from native English‐speaking countries, studies focused on vertebrates in terrestrial systems, and positive findings (Amano et al., 2023; Di Marco et al., 2017; Stahl et al., 2020; Wood, 2020). Although the problems with academic journals have been widely acknowledged across scientific disciplines, the responsibility to overcome barriers has largely been placed on authors rather than on publishers to work toward their removal. For example, many non‐native English speakers and non‐academic writers are encouraged by journals to seek professional English editing services to better conform to Western scientific styles and written standards (e.g., Hazen, 2016). Few conservation journals offer these editing services in‐house and even fewer offer these services for free. By asking authors to pay for additional services, publishers often place the burden of responsibility to overcome skill barriers on disadvantaged authors rather than working toward equity.

Although there has been momentum toward greater inclusivity in conservation research (Raymond et al., 2022) (e.g., broadening how journals credit author contributions [Cooke et al., 2022]), publishers may still be a bottleneck to the dissemination of conservation knowledge. Few conservation journals have made systemic changes to meet Fair Open Access Alliance standards, and authors often face further financial barriers if they wish or are required to make their research publicly available (Veríssimo et al., 2020). Many conservation journals offer a full or partial waiver for these article processing charges (APCs) for authors in low‐ and middle‐income countries. However, critics argue this does not go far enough to address inequity (Rouhi et al., 2022; Sanderson, 2023). Smith et al. (2021) found that waivers were ineffective at increasing the representation of low‐income authors. Such ineffectiveness may relate to poor communication surrounding waiver eligibility, waiver restrictions, or—less often discussed—negative perceptions toward waivers, which may be seen as patronizing by authors the journals are aiming to help (cOAlition S, 2024; Meagher, 2021). Others argue that APCs pose a barrier to many more authors who do not qualify for waivers, including authors from high‐income countries at small academic institutions or nonacademic organizations (Byrne, 2024; Frank et al., 2023).

Understanding to what extent journal characteristics, such as APCs, factor into journal choice and how this varies across author demographics and psychographics (psychological and behavioral variables) can therefore inform journal reform and facilitate more inclusive learning and knowledge exchange. We assessed preferences for certain journal attributes for researchers who have published in conservation science. Specifically, we assessed the interplay between 7 different journal attributes and how they affect an author's journal choice. We focused on published authors to gauge preferences in the current publishing landscape. Hence, we did not investigate hard barriers (i.e., barriers that prevent potential authors from publishing). Rather, we were interested in how these attributes affect choice for those already actively publishing. We subsequently contextualized the impact of current publishing decisions in conservation research and devised recommendations for conservation‐related journals on how to reduce challenges to publishing.

## METHODS

### Survey design

**FIGURE 1:**
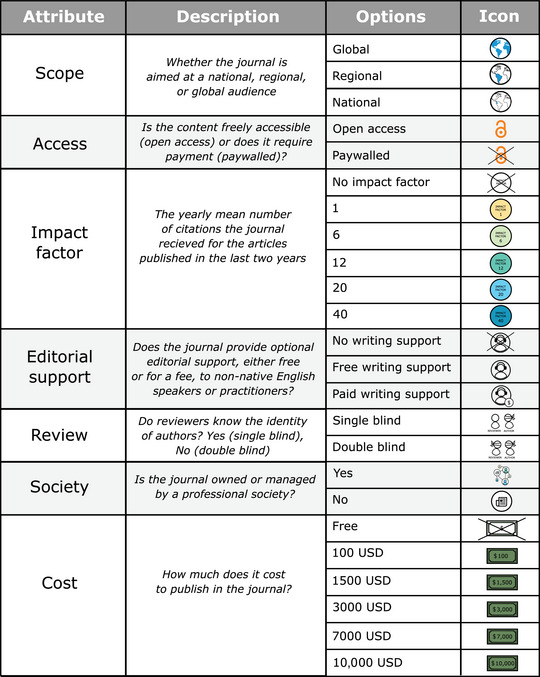
Attributes and levels of the discrete choice experiment investigating journal preference among conservation authors.

Our questionnaire consisted of a brief description of the survey background, questions related to respondents’ demographics, a discrete choice experiment focused on 7 key journal attributes, and a section for the respondent to rank conservation journal attributes (Appendix S1). In the first section of the survey, we confirmed whether the respondents had previously published in a peer‐reviewed, conservation‐related journal and, if so, how many conservation‐related papers they had published within the past year (in any author position). In the second section, we collected the respondent's demographic information: age, nationality, country of residence, and racial identity. Providing such information was voluntary. Asking demographic questions in this section of the survey may have primed authors to reflect on their demographic characteristics while completing the remainder of the survey (Hughes et al., 2016). However, it allowed us to collect demographic information on respondents who did not complete the survey or who submitted an incomplete survey. We determined which journal attributes to include in the discrete choice experiment following a workshop and online questionnaire where we asked attendants at the International Conference of Conservation Biology (ICCB) 2021 about how they choose where to publish (Appendix S2). From this preliminary data, we identified 7 main attributes that informed researchers publishing decisions (Figure 1). We used these 7 attributes to generate a discrete choice experiment with an orthogonal design generated in IBM SPSS 22.0. The initial choice alternatives were coupled, using a “shifted technique” (Louviere et al., 2000), into 16 trichotomous choices. The shifted technique introduces variation in the attribute levels between choice sets to better capture how decisions can vary depending on differences in the scenarios presented. We provided an opt‐out choice in the form of the choice “would not choose any of these journals” (choice d). In our study design, we referenced existing conservation and ecology journals to ensure attribute values reflected journals in the discipline.

The resultant data were analyzed using a multinomial logit model, and parameter estimates of the main effects were used as priors in a D‐efficient Bayesian design implemented in Ngene 1.0.1 to determine the final choice sets. We chose a multinomial logit model to analyze the resultant data because we asked respondents to choose among 4 options, including opt‐out. We specified a D‐efficient Bayesian prior design in which the parameter is described by its probability distribution rather than a fixed value to optimize the experiment design and improve precision (Rose & Bliemer, 2009). Parameter estimates of the main effects in the multinomial logit model were used as the priors. Using 500 Halton draws from normal prior distributions for each parameter, we compared the mean Bayesian D‐error of over 50,000 designs. The Bayesian D‐error quantifies how well a design extracts information from respondents in an experiment. We selected the model with the lowest error: 0.1606. We limited the number of scenarios to 12 to keep the discrete choice experiment design simple and to limit respondents’ cognitive burden. In the last section, we asked the respondents to rank the journal attributes from the most important to the least important.

### Data collection

We distributed the survey in 2 ways: directly through authors’ email addresses that we collected from published conservation articles and indirectly via communication platforms of conservation‐related institutions and organizations (newsletters, mailing lists, and social media platforms) (Appendix S3). Email addresses were collated for all authors (corresponding and noncorresponding authors) who published in 18 conservation‐related journals from 2010 to 2020 (Appendix S3) for whom contact information could be publicly obtained from recent publications or their affiliation webpage (*n* = 9994). We collected the data with SmartSurvey premium (www.smartsurvey.co.uk), an online survey software and questionnaire tool from 19 August to 3 November 2022. As an incentive for completing the survey, we offered respondents the opportunity to enter a raffle with the chance to win a 1‐year membership or a 3‐year membership to the Society for Conservation Biology (SCB) (three 1‐year and three 3‐year memberships were awarded). Only respondents who had published an article in a peer‐reviewed journal were considered in the analyses. Our study design received ethics clearance from the University of Oxford Central University Research Ethics Committee [R77648/RE001]. The data set associated with the study is archived with Zenodo (https://doi.org/10.5281/zenodo.8276263).

### Data analyses

We used different modeling approaches to investigate preferences for all respondents and distinct subpopulations of respondents. We used a multinomial logit model to evaluate the preferences of the entire sample of respondents. We used dummy coding to represent categorical variables with more than 2 categories (e.g., journal scope) in the model estimation (Appendix S4). This method is based on the assumption that preferences are homogenous across respondents. Because different author demographics may demonstrate different preferences among attributes, we were also interested in grouping respondents into distinct subpopulations observed in the total sampled population. To investigate this potential heterogeneity in choice, we employed 2 model specifications. First, we estimated a latent class model that allows for homogeneous finite classes. Each class represents a distinct segment of the respondent population, hereafter referred to as a segment (Boxall & Adamowicz, 2002). After a careful examination of the data, segment membership was explained by the inclusion of 3 socioeconomic variables: a dummy variable for income group (high income), age of respondents (age), and number of publications (number of publications). High and lower income were defined based on the World Bank income groups. For the latter, we combined middle‐income and low‐income countries. However, due to the representation of respondents, lower income countries predominantly represented those from middle‐income countries. The appropriate number of segments (e.g., the number of distinct subpopulations observed) was determined by examining a range of model statistics, including Akaike information criterion (AIC) and Bayesian information criterion (BIC).

Second, we used a mixed logit model to estimate willingness to pay and assumed all attributes followed a normal distribution (Balcombe et al., 2010). For all models to capture the neither responses, we included an alternative specific constant (ASC). When neither was selected, ASC assumed a value of 1, showing the utility gained from not selecting any of the available choice options. Both models are explained in detail in Appendix S5. Model comparison statistics (i.e., AIC, BIC) were generated for all model specifications examined (Appendix S6). Although the mixed logit model performed well, we focused our attention on the latent class model because it yields greater insights into respondent preferences. Full results for the mixed logit are reported in Appendices S7 and S8.

For the latent class models, we found that as we increased the number of segments from 3 to 4, the results started to become behaviorally unrealistic and unstable regarding the magnitude of the implied willingness to pay estimates. Thus, we report on a 3‐segment latent class model. With this model and the multinomial logit model (and for the mixed logit, see Appendix S7), we report willingness to pay for all attributes. Willingness to pay was the maximum price in US dollars a respondent was willing to pay for a particular attribute, such as a higher impact factor. For the multinomial logit model and the latent class model, we generated standard error estimates with the Wald method.

## RESULTS

A total of 1531 people responded to the survey. Of these, 1199 completed the survey and 1038 (86.57%) reported publishing in a conservation‐related study in a peer reviewed journal. On average, respondents published a mean of 3.28 papers (SD 5.26) over the previous year and were 40 years old (SD 11.31) (Appendix S9). Most respondents were from the United States (165 respondents, 15.90%), India (110 respondents, 10.60%), and the United Kingdom (84 respondents, 8.09%). Approximately half of the respondents (483 respondents, 46.53%) identified themselves as White Europeans, North Americans, Australians, or New Zealanders; 12.04% as South Asian; 8.67% as Southeast Asian, 7.61% as Latino, Latina, or Latinx; and 7.03% as Black African. We obtained 12,365 choice cards from 1038 respondents. Table 1 presents the main model results for the multinomial logit and the 3‐segment latent class model. For the latent class model, we also included the segment membership functions.

**TABLE 1:** Multinomial logit (MNL) and latent class model (LCM) estimates of utility function for each journal attribute in a model assessing responses to a discrete choice experiment examining author preferences when choosing where to publish conservation research.

		Latent class model segment^a^		
	MNL (SE)	Segment 1 (23.4%)	Segment 2 (30.9%)	Segment 3 (45.7%)
Attribute				
Alternative specific constant	−0.227 (0.077)^***^	1.037 (0.262)^***^	0.557 (0.184)^**^	−1.455 (0.131)^***^
Global journal scope	0.167 (0.052)^***^	−0.053 (0.181)	0.817 (0.161)^***^	0.547 (0.069)^***^
Regional journal scope	−0.001 (0.037)	−0.015 (0.146)	0.475 (0.094)^***^	0.237 (0.052)^***^
Access model^b^	0.357 (0.037)^***^	−0.177 (0.146)	0.980 (0.098)^***^	0.318 (0.058)^***^
Impact factor^c^	0.007 (0.001)^***^	0.004 (0.005)	0.035 (0.181)^***^	0.013 (0.002)^***^
No editorial support	−0.410 (0.042)^***^	−0.676 (0.173)^**^	0.101 (0.102)	−0.495 (0.058)^***^
Paid editorial support^d^	−0.546 (0.050)^***^	−1.492 (0.201)^***^	0.257 (0.122)^*^	−0.660 (0.069)^***^
Peer review model^e^	0.188 (0.028)^***^	0.725 (0.089)^***^	0.234 (0.074)^**^	0.226 (0.041)^***^
Society^f^	0.079 (0.026)^**^	0.290 (0.084)^**^	0.261 (0.067)^***^	0.054 (0.037)
Cost^g^	−0.252 (0.007)^***^	−0.390 (0.038)^***^	−1.109 (0.081)^***^	−0.169 (0.011)^***^
Segment membership functions				
Intercept		−1.236 (0.331)^***^	−0.552 (0.316)	
High income^h^		−0.812 (0.98)^***^	−0.660 (0.225)^**^	
Age^i^		0.025 (0.008)^**^	0.014 (0.008)	
Number of publications		−0.001 (0.014)	−0.017 (0.018)	

^a^ Segment: 1, more respondents from lower income countries and older respondents; 2, more respondents from lower income countries; 3, the reference class, represents younger respondents from high‐income countries. Significance levels: *p < 0.05, **p < 0.01, and ***p < 0.001.

^b^ Categorized as open access or paywalled (i.e., published under a subscription model).

^c^ Categorized as no impact factor, 1, 6, 12, 20, and 40.

^d^ Defined as paid writing support for non‐native English speakers and practitioners.

^e^ Categorized as single‐blind (author anonymized) and double‐blind (both author and reviewer are anonymous).

^f^ Categorized as not society‐based and society‐based.

^g^ Options provided (US$): free, 100, 1500, 3000, 7000, and 10,000.

^h^ Income group by country as defined by the World Bank (2022).

^i^ Categorized as 18–20, 21–29, 30–39, 40–49, 50–59, 60 or older, and prefer not to say.

Respondents ranked journal attributes from most to least important as follows: first, journal scope; second, whether a journal was open access; third, impact factor; fourth, cost; fifth, options for editorial support; sixth, whether a journal offered double‐blind review; and seventh, whether the journal was associated with a society. Although peer review model ranked low, willingness to pay estimates suggested it was important in journal choice (Appendix S7). A total of 312 respondents (30.65%) stated they ignored particular attributes when making their choices.

For both models (Table 1), the cost attribute was negative and always statistically significant. Segment 1 represented 23.4% of respondents. These respondents were older and from lower income countries given the segment membership function estimates. Lower income countries here included low‐income countries but predominantly represented middle‐income countries due to a lack of respondents from low‐income countries. The most statistically significant attributes for segment 1 were the availability of free editorial support (demonstrating a preference for free support over no support or paid support), a preference for journals offering double‐blind review, and journals associated with a society. For segment 2, the second largest group of respondents (30.9%), we found that more of the attributes were statistically significant than for segment 1. In particular, segment 2 respondents preferred journals with a global or regional scope, open access, and a high impact factor. Segment 2 respondents were the only segment to respond positively to the availability of paid editorial support. Given the segment membership function estimates for segment 2, respondents appeared to be younger and from lower income countries. Segment 3 had the largest proportion of respondents (45.7%), and all attributes were statistically significant, except whether a journal was associated with a society. Respondents in segment 3 had similar preferences to respondents in segment 2, except for editorial support. Segment 3 respondents avoided journals with no support or paid editorial support, comparable to segment 1 respondents. The class membership function was not estimated for segment 3 given the model identification restrictions. However, we inferred that respondents likely to be in this segment were relatively young (the sign for this parameter was positive for both segments 1 and 2), were from high‐income countries (the sign for this parameter is negative for both segments 1 and 2), and had more publications than respondents in the other segments (the sign for this parameter was negative for both segments 1 and 2). The results from the mixed logit for willingness to pay for our attributes (Appendix S8) revealed that willingness to pay was positively related to income, negatively related to age, and negatively but statistically weakly related to number of papers published. Willingness to pay estimates for the multinomial model and the latent class model are in Table 2.

**TABLE 2:** Conservation author willingness to pay for different attributes of academic journals.

		Latent class model (SE)		
Attribute	Multinomial logit model (SE)	Segment 1	Segment 2	Segment 3
Global journal scope	661.35^a^ (196.35)	NS	736.47^a^ (123.58)	3234.57^a^ (371.95)
Regional journal scope	NS	NS	428.02^a^ (76.19)	1400.18^a^ (297.55)
Open access	1413.12^a^ (131.67)	NS	883.30^a^ (90.73)	1882.13^a^ (279.51)
Impact factor	27.48^a^ (5.07)	NS	31.72^a^ (2.94)	77.80^a^ (10.61)
No editorial support	−1624.58^a^ (182.55)	−1734.79 (528.41)	NS	−2930.07^a^ (437.64)
Paid editorial support	−2161.80^a^ (222.92)	−3827.08 (746.84)	231.53 (108.87)	−3908.11^a^ (549.65)
Peer review model	743.96^a^ (111.92)	1861.33^a^ (289.81)	210.91 (71.06)	1335.67^a^ (252.94)
Society owned	314.15^a^ (102.99)	745.041 (231.27)	235.77^a^ (61.52)	NS

*Note*: Only statistically significant values in terms of influencing choice are provided (*p* < 0.05). All values are in US dollars. Segments defined in Table 1. Standard error generated using the Wald method.

Abbreviation: NS, not statistically significant.

^a^ Values with significance level at *p* < 0.01.

We found variation between the models in terms of the magnitude of willingness to pay and in the signs of the estimates (Table 2). All attributes for the multinomial logit and segment 3 of the latent class model had the same signs. However, segment 3 yielded by far the largest willingness to pay estimates compared with all other model results. Some results were similar between segments 2 and 3, which showed a preference for a broad journal scope, open access, and a high impact factor. However, the estimates for segment 3 were significantly bigger than that for segment 2: global or regional journal scope ($3235 and $1400, respectively), open access ($1882 and $883), and high impact factors ($78 and $32). None of these attributes were statistically significant for segment 1.

Peer review model was important for all segments, each yielding a positive willingness to pay estimate. Segment 1 yielded the largest estimate of $1861, relative to $1336 for segment 3. Respondents in segments 1 and 2 were also willing to pay for journals that were society owned, especially those in segment 1. For editorial support, willingness to pay estimates varied among segments. Segment 2 respondents showed a slight preference for journals offering paid editorial support. However, respondents in both segments 1 and 3 expressed negative preferences for no and paid editorial support options. Finally, when we compared the magnitude of our willingness to pay estimates with those of the mixed logit (Appendix S6), we saw that the mixed logit yielded on average lower estimates. The signs of the estimates in Appendix S7 were consistent with those reported in Table 2, but only journal impact factor ($14) and peer review ($795) yielded estimates that were similar in magnitude. These results indicated that we needed to treat our latent class model willingness to pay estimates as on the high side.

## DISCUSSION

Overall, we demonstrated that publishing preferences of conservation professionals are not homogenous across published authors. Cost and peer review model were the only 2 attributes to which all respondent segments responded consistently. Furthermore, we found that preferences for different journal attributes differed among World Bank income groups. These results highlight how different attributes may act as filters for different author demographics. We also demonstrated that journal preference is multifaceted because no one factor dictated journal choice. Impact factor has often been touted as a major driver of journal choice (Nicholas et al., 2014). However, despite ranking in the top 3 most influential attributes when self‐reporting, respondents were willing to pay little for high impact factors relative to other attributes, such as a global and regional scope or for open access. Unlike impact factor, many important attributes dictating journal choice are governed by the publisher. Therefore, publishers have the capacity to attract more authors and acknowledge their responsibility to uphold equitable publishing opportunities in conservation journals.

### Reducing the cost of access

Open Science, including free‐to‐read publishing, represents a positive step toward greater transparency and accessibility to scientific knowledge. Currently, however, open access represents a double‐edged sword for inclusion. Although open access mandates ensure researchers are obligated to make their research available to all, APC open access models can prevent research from being published at all for those who lack the resources to pay these fees. Much of the discussion regarding the “APC‐barrier” (Klebel & Ross‐Hellauer, 2023) has focused on authors in low‐ and middle‐income countries because they are likely to be the most restricted by APCs. However, our results showed that lower costs were preferred by authors across all demographics, including those in high‐income countries. Of the 18 conservation‐related journals we used to collate author email addresses, APCs ranged from $1632 (or $2040 for non‐society members) to $4600 (Appendix S10). Transformative agreements, including read‐and‐publish agreements (financial agreements between academic institutions and publishers whereby researchers can publish open access without charge), aim to shift the financial burden of publishing from authors to institutions. However, currently, these agreements reinforce existing inequities. At their best, read‐and‐publish agreements can restrict publishing options for authors by limiting which journals institutions will cover. At their worst, read‐and‐publish agreements limit who can contribute to the scientific conversation to only the most well‐funded, most‐resourced academic researchers (Debat & Babini, 2019). Neither these agreements nor waivers aim to change the status quo of commercial publishing models that prioritize profits over scientific dissemination (Byrne, 2024; Debat & Babini, 2019). Our findings showed that substantially reducing or preferentially removing costs would benefit all authors.

Nevertheless, hosting a journal requires infrastructure. Many societies outsource this requirement to commercial publishers, who in turn can help generate funds for the society to support activities, such as conferences, education and training, and future research, through publication fees. As such, many learned societies rely on their journal portfolio as vital revenue streams (Fyfe, 2023; Fyfe et al., 2017). However, this is at odds with many academic societies’ commitments to diversity, equity, and inclusion when publication fees act as a barrier to the very authors the society aims to support. Academic societies committed to addressing inequities in publishing in conservation may consider converting their journals to diamond open access models, such as with the *Edinburgh Journal of Botany*, or to initiatives such as *Peer Community in [Ecology]* and *SciPost* (PCI Ecology, 2023; SciPost Foundation, 2024). These models aim to provide a free alternative to traditional open access model journals.

*Edinburgh Journal of Botany* is published by the Royal Botanic Garden Edinburgh, a charity and nondepartmental UK Public Body. The journal was established in 1900 and moved to a diamond open access model in 2021 (RBGE, 2024). Similarly, *Chemical Science* is a diamond open access journal for the Royal Society of Chemistry, which received 6980 submissions in 2023 and has a 5‐year impact factor of 8.6 (RSC, 2024). The journal has options for both single‐ and double‐blind review, fast processing times, and optional transparent peer review (e.g., where the reviewer comments and responses are published alongside the final article) (RSC, 2024). Both represent different pathways for conservation societies to adopt free‐to‐publish–free‐to‐read publishing models. In doing so, societies can ensure it is the quality of the peer review, not the price to publish, that dictates the perception of quality in conservation literature.

Currently, most society‐owned diamond open access journals rely on society membership fees to cover operational costs (Bosman et al., 2021). We found younger respondents in high‐income countries were less likely to prioritize society‐owned journals. As early as 2008, concerns were raised over the decline of young professionals joining academic societies, such as the SCB (Grajal, 2009; Schwartz et al., 2008). Grajal (2009) argued academic societies need to explore ways to increase their value for younger conservation professionals, and our research indicates an opportunity to do so specifically in the publishing domain.

### Promoting equitable peer review

Several studies have demonstrated how single‐blind reviews can offer advantages to authors from high‐income, English‐speaking countries across biological science journals, including in *Functional Ecology*, which is likely due to prestige bias (Fox et al., 2023; Smith et al., 2023) (e.g., where reviewers expect work from certain countries, institutions, or individuals to be of higher quality). Despite this, Smith et al. (2023) found only 15.9% of 541 biological science journals have double‐blind review, including universal double‐blind review (e.g., *Conservation Biology*) or optional double‐blind review (e.g., *Nature Ecology and Evolution*). We found all author segments had a preference for double‐blind over single‐blind peer review. Previous research in the medical literature (Parmanne et al., 2023) also shows transitioning to double‐blind review does not affect a reviewer's willingness to review. Therefore, introducing double‐blind review is a cost‐neutral way for journals to attract new authors, while at the same time working toward reducing unconscious (or conscious) bias in peer review.

Anonymizing work merely represents an initial step in mitigating bias in the peer review process. Anonymized authors may still face discrimination from reviewers and editors should they diverge from predetermined language and style criteria, irrespective of the scientific merit of their work. Nevertheless, despite an increased likelihood of journal rejections, Amano et al. (2023) found many authors do not or cannot access paid, professional editing services, particularly those from low‐ and middle‐income countries. Our results indicate that 2 of the 3 segments actively avoided journals with the option to pay more for editorial support, even when compared with journals that offer no support. More concerning still is the insistence that authors specifically seek collaborations with native English speakers to ensure they produce “high‐quality research articles” (Balan, 2021). Needless to say, collaboration should be born from more than someone's first language, and such comments are an insult to the groundbreaking work undertaken by non‐native English speakers around the world. If journals insist that publications be written in English (but see Amano et al. [2021, 2023] and Chowdhury et al. [2022]), it should be their responsibility to support authors rather than placing all the burden on non‐native English‐speaking authors. Offering such assistance would in return aid reviewers, whose voluntary role and expertise should be to assess the quality of the research. *Conservation Biology* and *Conservation Science and Practice* support authors by offering an alternative strategy through their Publication Partner Program (SCB, 2023). This free initiative invites authors to partner with an experienced volunteer who can help with manuscript revisions, aiming to improve the likelihood of publication. Such peer‐support strategies acknowledge systemic barriers and provide training and support to those who are disadvantaged by the current publishing environment. However, we found that certain respondents did not exhibit a preference for free support over no editorial support. Thus, more research is needed to ensure support schemes are meeting the needs of those they aim to help.

### Those missing from the conversation

We found that all segments published similar numbers of publications over the study period (August 2021 to November 2022), suggesting journal preferences did not affect how many publications our respondents were able to publish among segments. Yet, given that just 3 countries represented over a third of our respondents and almost half of the respondents identified as White Europeans, North Americans, Australians, or New Zealanders, our sample suggests barriers are hindering a more diverse representation of authors from publishing in the first instance. Our sample was largely dominated by respondents from high‐income and upper‐middle‐income countries (51.8% and 20.1% of respondents, respectively); few respondents were from low‐income countries (2.0%) (Appendix S9). Consequently, our sample does not capture hard barriers to publishing faced by authors in lower‐middle‐ and low‐income countries, be it related to costs, review bias, or other factors. Subsequent research should survey current authors and potential authors to determine the publishing preferences and barriers more comprehensively for authors in these regions. This may include assessing differences in how willingness to pay is expressed differently across different demographics (i.e., cannot pay vs. choose not to pay). Future research could also explore how other dimensions of an author's identity affect journal choices, such as gender, discipline (e.g., social science vs. natural science vs. humanities), industry (e.g., between academia and nonacademic sectors), focal taxa or ecosystem, career stage, and tenure (Griffiths & Dos Santos, 2012; Maas et al., 2021; Teel et al., 2018). There may also be additional journal attributes, such as perceptions of peer review quality, that were not captured by the initial workshop and questionnaire at ICCB. Such attributes may be associated with certain demographics that were not well represented by those at ICCB, such as peer review speed for early‐career researchers (Nguyen et al., 2015). Future research could explore how choice is affected by measurable or perceived differences in the review process among journals.

### How perceptions of research value affect conservation

Although regional‐ or local‐scale research is often most informative for on‐the‐ground conservation practitioners (Calver et al., 2010; Stergiou & Tsikliras, 2006), academic career incentives do not necessarily align with impact outside of academia (Rigby et al., 2015). Authorship in journals considered high ranking or prestigious is perceived as important for a researcher's career progression (Nicholas et al., 2017; Rigby et al., 2015), and many of the most highly ranked conservation journals now prioritize studies with a broad geographical or taxonomic scope. These large‐scale studies are often beneficial for identifying priority areas of future research or intervention. Cultivating research deemed applicable for a broader audience is also beneficial to the publisher because it increases the likelihood of citations and thus the currency used to bolster the perceived importance and legitimacy of a journal and therefore the likelihood of future submissions. This perpetuates a cycle of science that is not necessarily engaged with applied conservation needs. In this way, publishers run the risk of creating a dilemma for researchers—a trade‐off between safeguarding their career and securing funding versus contributing to local conservation efforts. Learned societies have a unique opportunity to capitalize on their authority and credibility to challenge this perception of value. Unlike new independent journals, journals run by learned societies do not rely on impact factor, and thus citations, to demonstrate their scientific legitimacy. Free of this constraint, such journals have more freedom to find new ways to recognize meaningful contributions to science beyond the number of citations—for example, providing publishing opportunities that prioritize science that informs conservation impact (but potentially within a limited scope) over highly citable, global reviews with limited conservation applications. Although both are valuable contributions to the academic literature, there are many publishing opportunities for the latter, but few prestigious journal portfolios offer the former (but see *Conservation Science and Practice* [SCB, 2024]).

We outlined several ways in which journals can affect the conservation literature and future research directions. Given our findings, we make several recommendations for publishers that would improve equity, diversity, and inclusion in conservation publishing and ultimately benefit conservation science. We recognize systemic issues in academic publishing go beyond the conservation literature. In all fields, commercial publishers lack incentives to deviate from a publishing landscape that fails authors and readers when they seek to benefit from the substantial profits it generates (Van Noorden, 2013). In a time when the market is becoming increasingly saturated by predatory publishing practices, learned societies are presented with the opportunity to overhaul how they disseminate and value science. Conservation is distinct from other sciences due to its interdisciplinary nature, its close ties to policy and management, and the need to be dynamic in the face of changing environmental conditions and public perceptions. Therefore, conservation literature benefits more than most by having the largest diversity of voices at the table. Finally, we acknowledge that publishing is the last stage in the research pipeline. Many people will have already been excluded from the publishing process in the research planning and execution stages. Collectively, we are all responsible for improving equity, diversity, and inclusion across the entire research process.

## AUTHOR CONTRIBUTIONS

**Natalie Yoh**: Methodology; duration curation; investigation; writing—original draft preparation; writing—reviewing and editing. **Mukhlish Jamal Musa Holle**: Methodology; duration curation; investigation; writing—reviewing and editing. **Jasmin Willis**: Data curation and project administration. **Lauren F. Rudd**: Data curation; project administration; writing—reviewing and editing. **Iain M. Fraser**: Methodology; formal analysis; writing—reviewing and editing. **Diogo Veríssimo**: Conceptualization; methodology; investigation; funding acquisition; supervision; writing—reviewing and editing.

## Supporting information

Supporting Information

## References

[cobi14369-bib-0001] Amano, T. , Berdejo‐Espinola, V. , Christie, A. P. , Willott, K. , Akasaka, M. , Báldi, A. , Berthinussen, A. , Bertolino, S. , Bladon, A. J. , Chen, M. , Choi, C.‐Y. , Kharrat, M. B. D. , Oliveira, L. G. D. , Farhat, P. , Golivets, M. , Aranzamendi, N. H. , Jantke, K. , Kajzer‐Bonk, J. , Aytekin, M. Ç. K. , … Sutherland, W. J. (2021). Tapping into non‐English‐language science for the conservation of global biodiversity. PLoS Biology, 19, Article e3001296. 34618803 10.1371/journal.pbio.3001296 PMC8496809

[cobi14369-bib-0002] Amano, T. , Ramírez‐Castañeda, V. , Berdejo‐Espinola, V. , Borokini, I. , Chowdhury, S. , Golivets, M. , González‐Trujillo, J. D. , Montaño‐Centellas, F. , Paudel, K. , White, R. L. , & Veríssimo, D. (2023). The manifold costs of being a non‐native English speaker in science. PLoS Biology, 21, Article e3002184. 37463136 10.1371/journal.pbio.3002184 PMC10353817

[cobi14369-bib-0003] Balan, S. (2021). English as the language of research: But are we missing the mark? Exploratory Research in Clinical and Social Pharmacy, 3, Article 100043. 35480598 10.1016/j.rcsop.2021.100043 PMC9032012

[cobi14369-bib-0056] Balcombe, K. , Fraser, I. , & Falco, S. D. (2010). Traffic lights and food choice: A choice experiment examining the relationship between nutritional food labels and price. Food Policy, 35(3), 211–220. 10.1016/j.foodpol.2009.12.005

[cobi14369-bib-0004] Bosman, J. , Frantsvåg, J. E. , Kramer, B. , Langlais, P.‐C. , & Proudman, V. (2021). OA diamond journals study. Part 1: Findings . Zenodo. https://zenodo.org/records/4558704

[cobi14369-bib-0005] Boxall, P. C. , & Adamowicz, W. L. (2002). Understanding heterogeneous preferences in random utility models: A latent class approach. Environmental and Resource Economics, 23, 421–446.

[cobi14369-bib-0006] Byrne, L. B. (2024). Authors from wealthy countries cannot all pay publishing fees. Nature, 625, 450–450. 10.1038/d41586-024-00116-6 38228791

[cobi14369-bib-0007] Calver, M. , Wardell‐Johnson, G. , Bradley, S. , & Taplin, R. (2010). What makes a journal international? A case study using conservation biology journals. Scientometrics, 85, 387–400.

[cobi14369-bib-0008] Chowdhury, S. , Gonzalez, K. , Aytekin, M. Ç. K. , Baek, S.‐Y. , Bełcik, M. , Bertolino, S. , Duijns, S. , Han, Y. , Jantke, K. , Katayose, R. , Lin, M.‐M. , Nourani, E. , Ramos, D. L. , Rouyer, M.‐M. , Sidemo‐Holm, W. , Vozykova, S. , Zamora‐Gutierrez, V. , & Amano, T. (2022). Growth of non‐English‐language literature on biodiversity conservation. Conservation Biology, 36, Article e13883. 34981574 10.1111/cobi.13883 PMC9539909

[cobi14369-bib-0009] cOAlition S . (2024). Developing a globally fair pricing model for open access academic publishing . https://www.coalition‐s.org/developing‐a‐globally‐fair‐pricing‐model‐for‐open‐access‐academic‐publishing/

[cobi14369-bib-0010] Cooke, S. J. , Michaels, S. , Nyboer, E. A. , Schiller, L. , Littlechild, D. B. R. , Hanna, D. E. L. , Robichaud, C. D. , Murdoch, A. , Roche, D. , Soroye, P. , Vermaire, J. C. , Nguyen, V. M. , Young, N. , Provencher, J. F. , Smith, P. A. , Mitchell, G. W. , Avery‐Gomm, S. , Davy, C. M. , Buxton, R. T. , … Auld, G. (2022). Reconceptualizing conservation. PLoS Sustainability and Transformation, 1, Article e0000016.

[cobi14369-bib-0011] Debat, H. , & Babini, D. (2019). Plan S in Latin America: A precautionary note. PeerJ Preprints, 7, Article e27834v2.

[cobi14369-bib-0012] Di Marco, M. , Chapman, S. , Althor, G. , Kearney, S. , Besancon, C. , Butt, N. , Maina, J. M. , Possingham, H. P. , Rogalla von Bieberstein, K. , Venter, O. , & Watson, J. E. M. (2017). Changing trends and persisting biases in three decades of conservation science. Global Ecology and Conservation, 10, 32–42.

[cobi14369-bib-0013] Fox, C. , Meyer, J. , & Aimé, E. (2023). Double‐blind peer review affects reviewer ratings and editor decisions at an ecology journal. Functional Ecology, 37, 1144–1157.

[cobi14369-bib-0014] Frank, J. , Foster, R. , & Pagliari, C. (2023). Open access publishing—Noble intention, flawed reality. Social Science & Medicine, 317, Article 115592. 36481722 10.1016/j.socscimed.2022.115592

[cobi14369-bib-0015] Fyfe, A. (2023). Do journals need societies, and do societies need journals? Recenti Progressi in Medicina, 114, 154–156. 36815418 10.1701/3981.39639

[cobi14369-bib-0016] Fyfe, A. , Coate, K. , Curry, S. , Lawson, S. , Moxham, N. , & Røstvik, C. M. (2017). Untangling academic publishing: A history of the relationship between commercial interests, academic prestige and the circulation of research. University of St Andrews.

[cobi14369-bib-0017] Grajal, A. (2009). Increasing the value of SCB membership for young professionals. Conservation Biology, 23, 246–247. 10.1111/j.1523-1739.2008.01199.x 19323676

[cobi14369-bib-0018] Griffiths, R. A. , & Dos Santos, M. (2012). Trends in conservation biology: Progress or procrastination in a new millennium? Biological Conservation, 153, 153–158.

[cobi14369-bib-0019] Hall, C. M. , & Page, S. J. (2015). Following the impact factor: Utilitarianism or academic compliance? Tourism Management, 51, 309–312.

[cobi14369-bib-0020] Hazen, B. T. (2016). Overcoming basic barriers to publishing research. The International Journal of Logistics Management, 27(1). 10.1108/IJLM-12-2015-0226

[cobi14369-bib-0021] Hughes, J. , Camden, A. , & Yangchen, T. (2016). Rethinking and updating demographic questions: Guidance to improve descriptions of research samples. Psi Chi Journal of Psychological Research, 21, 138–151.

[cobi14369-bib-0022] Klebel, T. , & Ross‐Hellauer, T. (2023). The APC‐barrier and its effect on stratification in open access publishing. Quantitative Science Studies, 4, 22–43.

[cobi14369-bib-0023] Louviere, J. J. , Hensher, D. A. , & Swait, J. D. (2000). Stated choice methods: Analysis and applications. Cambridge University Press.

[cobi14369-bib-0024] Maas, B. , Pakeman, R. J. , Godet, L. , Smith, L. , Devictor, V. , & Primack, R. (2021). Women and Global South strikingly underrepresented among top‐publishing ecologists. Conservation Letters, 14, Article e12797.

[cobi14369-bib-0025] Meagher, K. (2021). Introduction: The politics of open access—Decolonizing research or corporate capture? Development and Change, 52, 340–358.

[cobi14369-bib-0026] Medina‐Franco, J. L. , & López‐López, E. (2022). The essence and transcendence of scientific publishing. Frontiers in Research Metrics and Analytics, 7, Article 822453. 35252740 10.3389/frma.2022.822453 PMC8888534

[cobi14369-bib-0027] Nguyen, V. M. , Haddaway, N. R. , Gutowsky, L. F. G. , Wilson, A. D. M. , Gallagher, A. J. , Donaldson, M. R. , Hammerschlag, N. , & Cooke, S. J. (2015). How long is too long in contemporary peer review? Perspectives from authors publishing in conservation biology journals. PLoS ONE, 10, Article e0132557. 26267491 10.1371/journal.pone.0132557 PMC4533968

[cobi14369-bib-0028] Nicholas, D. , Rodríguez‐Bravo, B. , Watkinson, A. , Boukacem‐Zeghmouri, C. , Herman, E. , Xu, J. , Abrizah, A. , & Świgoń, M. (2017). Early career researchers and their publishing and authorship practices. Learned Publishing, 30, 205–217. 10.1371/journal.pone.0281058 PMC9931124 36791119

[cobi14369-bib-0029] Nicholas, D. , Watkinson, A. , Volentine, R. , Allard, S. , Levine, K. , Tenopir, C. , & Herman, E. (2014). Trust and Authority in Scholarly Communications in the Light of the Digital Transition: Setting the scene for a major study. Learned Publishing, 27, 121–134.

[cobi14369-bib-0030] Oshiro, J. , Caubet, S. L. , Viola, K. E. , & Huber, J. M. (2020). Going Beyond “not enough time”: Barriers to preparing manuscripts for academic medical journals. Teaching and Learning in Medicine, 32, 71–81. 31530189 10.1080/10401334.2019.1659144 PMC6904427

[cobi14369-bib-0031] Parmanne, P. , Laajava, J. , Järvinen, N. , Harju, T. , Marttunen, M. , & Saloheimo, P. (2023). Peer reviewers’ willingness to review, their recommendations and quality of reviews after the Finnish Medical Journal switched from single‐blind to double‐blind peer review. Research Integrity and Peer Review, 8, Article 14. 37876004 10.1186/s41073-023-00140-6 PMC10598992

[cobi14369-bib-0032] PCI Ecology . (2023). What is PCI Ecology? Peer Community. https://ecology.peercommunityin.org/about/about

[cobi14369-bib-0033] Prasojo, L. D. P. , Habibi, A. , Sofwan, M. , Yaakob, M. F. M. , Mukminin, A. , & Yuliana, L. (2019). Barriers to publication: Stories of Ph.D. students from Malaysian Universities. Library Philosophy and Practice, 2019, Article 3567.

[cobi14369-bib-0034] Raymond, C. M. , Cebrián‐Piqueras, M. A. , Andersson, E. , Andrade, R. , Schnell, A. A. , Battioni Romanelli, B. , Filyushkina, A. , Goodson, D. J. , Horcea‐Milcu, A. , Johnson, D. N. , Keller, R. , Kuiper, J. J. , Lo, V. , López‐Rodríguez, M. D. , March, H. , Metzger, M. , Oteros‐Rozas, E. , Salcido, E. , Sellberg, M. , … Wiedermann, M. M. (2022). Inclusive conservation and the Post‐2020 Global Biodiversity Framework: Tensions and prospects. One Earth, 5, 252–264.

[cobi14369-bib-0035] Rigby, D. , Burton, M. , & Lusk, J. L. (2015). Journals, preferences, and publishing in agricultural and environmental economics. American Journal of Agricultural Economics, 97, 490–509.

[cobi14369-bib-0036] Rose, J. M. , & Bliemer, M. C. J. (2009). Constructing efficient stated choice experimental designs. Transport Reviews, 29, 587–617.

[cobi14369-bib-0037] Rouhi, S. , Beard, R. , & Brundy, C. (2022). Left in the cold: The failure of APC waiver programs to provide author equity. Science Editor, 45, 5–13.

[cobi14369-bib-0038] Royal Botanic Garden Edinburgh (RBGE) . (2024). Edinburgh Journal of Botany . https://journals.rbge.org.uk/ejb/about

[cobi14369-bib-0039] Royal Society of Chemistry (RSC) . (2024). Chemical science . https://www.rsc.org/journals‐books‐databases/about‐journals/chemical‐science/

[cobi14369-bib-0040] Sanderson, K. (2023). Who should pay for open‐access publishing? APC alternatives emerge. Nature, 623, 472–473.37964063

[cobi14369-bib-0041] Schwartz, M. W. , Hunter, M. L. , & Boersma, P. D. (2008). Scientific societies in the 21st century: A membership crisis. Conservation Biology, 22, 1087–1089. 18954335 10.1111/j.1523-1739.2008.01059.x

[cobi14369-bib-0042] SciPost Foundation . (2024). SciPost . https://scipost.org/about#GOA

[cobi14369-bib-0043] Smith, A. C. , Merz, L. , Borden, J. B. , Gulick, C. K. , Kshirsagar, A. R. , & Bruna, E. M. (2021). Assessing the effect of article processing charges on the geographic diversity of authors using Elsevier's “Mirror Journal” system. Quantitative Science Studies, 2, 1123–1143.

[cobi14369-bib-0044] Smith, O. M. , Davis, K. L. , Pizza, R. B. , Waterman, R. , Dobson, K. C. , Foster, B. , Jarvey, J. C. , Jones, L. N. , Leuenberger, W. , Nourn, N. , Conway, E. E. , Fiser, C. M. , Hansen, Z. A. , Hristova, A. , Mack, C. , Saunders, A. N. , Utley, O. J. , Young, M. L. , & Davis, C. L. (2023). Peer review perpetuates barriers for historically excluded groups. Nature Ecology and Evolution, 7, 512–523. 36914773 10.1038/s41559-023-01999-w

[cobi14369-bib-0045] Society for Conservation Biology (SCB) . (2023). Publication partner program . https://conbio.onlinelibrary.wiley.com/page/journal/15231739/homepage/publication_partner_program.htm

[cobi14369-bib-0046] Society for Conservation Biology (SCB) . (2024). Overview: Conservation science and practice . https://conbio.onlinelibrary.wiley.com/hub/journal/25784854/homepage/overview

[cobi14369-bib-0047] Stahl, K. , Lepczyk, C. A. , & Christoffel, R. A. (2020). Evaluating conservation biology texts for bias in biodiversity representation. PLoS ONE, 15, Article e0234877. 32649672 10.1371/journal.pone.0234877 PMC7351170

[cobi14369-bib-0048] Stergiou, K. , & Tsikliras, A. (2006). Underrepresentation of regional ecological research output by bibliometric indices. Ethics in Science and Environmental Politics, 6, 15–17.

[cobi14369-bib-0049] Stirling, A. , & Burgman, M. A. (2021). Strengthening conservation science as a crisis discipline by addressing challenges of precaution, privilege, and individualism. Conservation Biology, 35, 1738–1746. 34405462 10.1111/cobi.13809

[cobi14369-bib-0050] Teel, T. L. , Anderson, C. B. , Burgman, M. A. , Cinner, J. , Clark, D. , Estévez, R. A. , Jones, J. P. G. , McClanahan, T. R. , Reed, M. S. , Sandbrook, C. , & St John, F. A. V. (2018). Publishing social science research in Conservation Biology to move beyond biology. Conservation Biology, 32, 6–8. 29314313 10.1111/cobi.13059

[cobi14369-bib-0051] Tomkins, A. , Zhang, M. , & Heavlin, W. D. (2017). Reviewer bias in single‐ versus double‐blind peer review. Proceedings of the National Academy of Sciences of the United States of America, 114, 12708–12713. 29138317 10.1073/pnas.1707323114 PMC5715744

[cobi14369-bib-0052] Van Noorden, R. (2013). Open access: The true cost of science publishing. Nature, 495, 426–429. 23538808 10.1038/495426a

[cobi14369-bib-0053] Veríssimo, D. , Pienkowski, T. , Arias, M. , Cugnière, L. , Doughty, H. , Hazenbosch, M. , de Lange, E. , Moskeland, A. , & Grace, M. (2020). Ethical publishing in biodiversity conservation science. Conservation & Society, 18, 220–225.

[cobi14369-bib-0054] Wood, K. A. (2020). Negative results provide valuable evidence for conservation. Perspectives in Ecology and Conservation, 18, 235–237.

