# Supplementary material for: Understanding author choices in the current conservation publishing landscape
Source: Conserv Biol. 2024 Sep 3;39(2):e14369. doi: 10.1111/cobi.14369 (PMC11959313; doi:10.1111/cobi.14369)
Supplement: Supplementary file 1 — Supporting Information [file COBI-39-e14369-s001.pdf]

Supporting Information

Appendix 1. Example of the survey including the background information provided to respondents of the Discrete Choice Experiment

Background information

1. What is the aim of this study?

You have been invited to complete the survey below as a conservation researcher. The study aims to identify the preferences of different groups (e.g. social or ethnic groups) when choosing conservation research publishers, in order to identify strategies to improve inclusivity in conservation publishing. This study is supported by the Society for Conservation Biology (SCB). The Principal Researcher is Diogo Veríssimo, who is attached to the Department of Zoology at the University of Oxford as a Postdoctoral research fellow.

2. What do I need to do?

You are being asked to complete a demographic survey and to respond to several choices about conservation publication. The survey should take approximately 10 minutes to complete. Please note that the survey responses are anonymous. By submitting your response to this survey, you are consenting that your data can be anonymously used for the study.

3. Do I have to take part?

No. Please note that participation is voluntary. If you do decide to take part, you may withdraw at any point for any reason before submitting your answers by pressing the ‘Exit’ button/closing the browser.

4. How will my data be used?

All data will be kept in a password-protected file within the University of Oxford’s secure network and will not be shared with any third parties. We will keep all data records from the survey responses for three years post the publication of this research. Your contact details were obtained from publicly available sources, but will be deleted when the study has been completed.

5. Who has reviewed this study?

This project has been reviewed by, and received ethics clearance through, a subcommittee of the University of Oxford Central University Research Ethics Committee [R77648/RE001].

6. Who do I contact if I have a concern or I wish to complain?

If you have a concern about any aspect of this study, please speak to Diogo Veríssimo (diogo.gasparverissimo@zoo.ox.ac.uk), and we will do our best to answer your query. We will acknowledge your concern within 10 working days and give you an indication of how it will be dealt with. If you remain unhappy or wish to make a formal complaint, please contact the Chair of the Medical Sciences Interdivisional Research Ethics Committee at the University of Oxford who will seek to resolve the matter as soon as possible: Email: ethics@medsci.ox.ac.uk; Address: Research Services, University of Oxford, Boundary Brook House, Churchill Drive, Headington, OX3 7GB.

Please contact Diogo Veríssimo (diogo.gasparverissimo@zoo.ox.ac.uk) for more information about this study.

Demographic survey

1. If you have read the information above and agree to participate with the understanding that the data you submit will be processed accordingly, please tick the box below to start. *
   ☐ Yes, I agree to take part.
2. Please note that you may only participate in this survey if you are 18 years of age or over. *
   ☐ I certify that I am 18 years of age or over.
3. Have you ever published a conservation-related study in a peer-reviewed journal? *
   ☐ Yes
   ☐ No
4. If you have published a conservation-related paper in a peer-reviewed journal, how many papers did you publish last year? (*Fill with number*)
5. What is your age? *
   ☐ 18-20
   ☐ 21-29
   ☐ 30-39
   ☐ 40-49
   ☐ 50-59
   ☐ 60 or older
   ☐ Prefer not to say
6. What is your nationality? The question allows for multiple options to be selected *
   Tick box options including "Prefer not to say" and "Unknown".
7. What is your country of residence * [Dropdown menu]
   Dropdown menu including "Prefer not to say".

8. How would you best describe your race? This question allows for multiple options to be selected *
Tick box options including "No response" and "Other option not listed here".

Introduction to Choice Experiment

You will be now asked to make a series of choices between academic journals with different characteristics. Please answer as if you were considering where to publish your next paper. These characteristics are:

Attribute	Description	Options	Icon
Scope	*Whether the journal is aimed at a national, regional, or global audience*	Global	
		Regional	
		National	
Access	*Is the content freely accessible (open access) or does it require payment (paywalled)?*	Open access	
		Paywalled	
Impact factor	*The yearly mean number of citations the journal recieved for the articles published in the last two years*	No impact factor	
		1	
		6	
		12	
		20	
		40	
Editorial support	*Does the journal provide optional editorial support, either free or for a fee, to non-native English speakers or practitioners?*	No writing support	
		Free writing support	
		Paid writing support	
Review	*Do reviewers know the identity of authors? Yes (single blind), No (double-blind)*	Single blind	
		Double blind	
Society	*Is the journal owned or managed by a professional society?*	Yes	
		No	
Cost	*How much does it cost to publish in the journal?*	Free	
		100 USD	$100
		1500 USD	$1,500
		3000 USD	$3,000
		7000 USD	$7,000
		10000 USD	$10,000

If none of the options provided is acceptable, you also have the option to not choose.

Choices

9-20. Imagine you have a manuscript ready for publication and you are about to submit your manuscript. There are three options of journal publishers with different services as follows; which alternative would you choose?

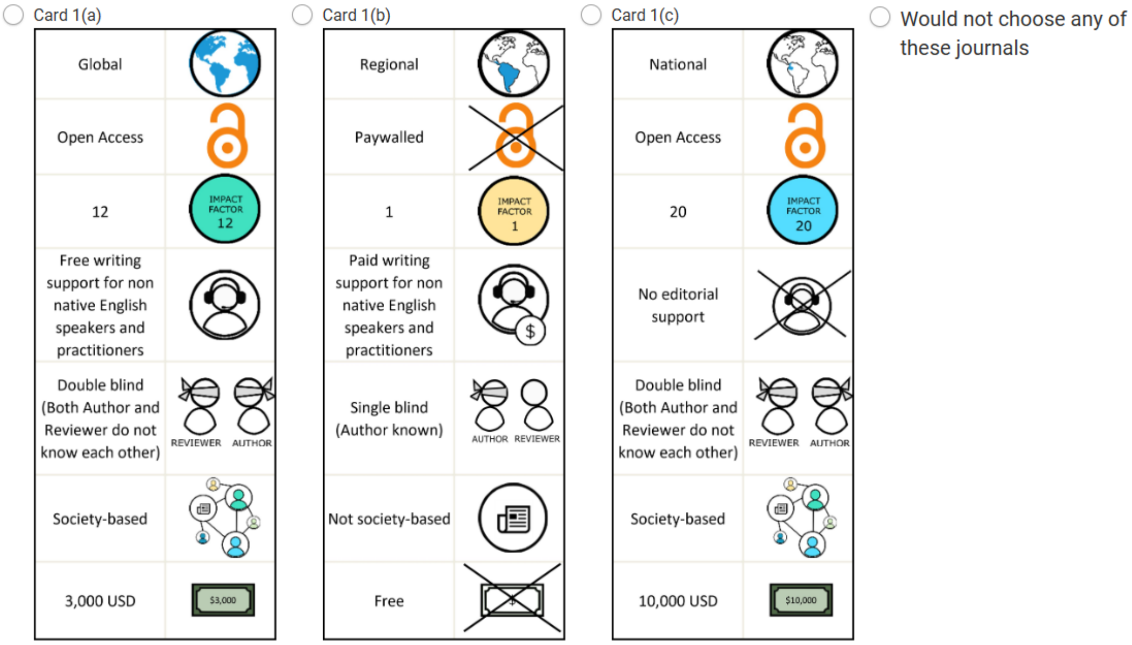

Example of choice card design

Attribute preference

21. Please rank following attributes from 7 (the most important attribute) to 1 (the least important attribute)

☐ Scope

☐ Access

☐ Impact factor

☐ Editorial support

☐ Review

☐ Society

☐ Cost

22. Have you ignored attributes?

☐ Yes

☐ No

Win a membership to the Society for Conservation Biology!

We are offering all survey respondents the chance to win a membership to the Society for Conservation Biology. We have three 1-year memberships and three 3-year memberships available. If you would like to be considered, please click the link below. If you do not wish to enter the raffle, please select 'Finish survey' to submit your response.

RETRACTED FOR REVIEW

If you have any questions, please speak to RETRACTED FOR REVIEW (RETRACTED FOR REVIEW).

Appendix 2. Summary of preliminary surveys at the International Conference of Conservation Biology

To identify important journal attributes to include in the Discrete Choice Experiment, we conducted two surveys during the International Conference of Conservation Biology (2021). Firstly, we hosted a one-hour workshop “Barriers and Opportunities in Academic Publishing” where we divided the 12 attendees (excluding facilitators) into three breakout rooms and each group was asked to discuss two questions, 1) how do you choose where to publish, and 2) what can SCB journals change to better serve the conservation science community? We then compiled the responses from each breakout room. Secondly, we invited conference attendees to complete an online survey where we asked the same two questions along with additional questions, including country of residence and previous experience in publishing conservation research. In total, we received 86 respondents from 33 countries. Seventy-six percent of respondents reported they had previously published in a peer reviewed journal. Topic relevance (30% of respondents), impact factor/reputation (29%), cost (21%), readership (5%), and whether the journal was open access (5%) were the most common factors identified from these surveys. Career type, colleague recommendations, discrimination during the review process, and conservation impact were also identified less frequently.

Appendix 3. Information used to invite survey participants. (a) Conservation-related journals used to collect author emails from publications in 2010 and 2020. (b) Conservation-related institutions and organisations whose communication platforms (e.g. newsletters, mailing lists, and social media platforms) were used to advertise the survey more broadly.

(a) Conservation-related journals

Aquatic Conservation:
Marine & Freshwater Ecosystems

Biodiversity and Conservation

Biological Conservation

Conservation Biology

Conservation Letters

Conservation Physiology

Conservation Science and Practice

Diversity and Distributions

Ecological Engineering

Environmental Conservation

Global Ecology and Conservation

Human-Wildlife Interactions

Journal for Nature Conservation

Journal of Applied Ecology

Landscape Ecology

Nature Conservation

Oryx

Wildlife Society Bulletin

(b) Communication channels

Society for Conservation Biology

Durrell Institute of Conservation and Ecology

Association for Tropical Biology and Conservation

Conservation Leadership Program

Interdisciplinary Center for Conservation Science

The Smith Fellowship

The Rufford Foundation

The Student Conference in Conservation Science (SCCS):

Bangalore, Cambridge, Australia

The International Union for Conservation of Nature (IUCN):

- Knowledge Unit
- Species Survival Commission
- Commission on Education & Communication
- Other commissions

National Geographic Explorers

Cambridge Conservation Initiative

Appendix 4: Dummy coding used in model estimation to evaluate journal preferences.

**Variable**	**Type**
Journal Scope Global	Dummy
Journal Scope Regional	Dummy
Journal Scope Local	Dummy*
Access Model	Dummy
Impact Factor	Score
Edit No	Dummy
Edit Paid	Dummy
Edit Free	Dummy*
Peer Review Model	Dummy
Society	Dummy
Cost	Monetary

Note: * excluded dummy level

Appendix 5: Econometric Model Specifications

Our survey data is used to model a utility function representing respondent preferences over a set of choices. The utility function depends on the attributes of each choice made by respondent *i (i=1,…,N)*. Let x_{ijt} be a k×1 vector of attributes presented to respondent *i* such that they select alternative *j* (*j*=1,…,*J*) in choice situation *t* (*t*=1,…,*T*). Assume U_{ijt} is the utility respondent *i* attains from x_{ijs} such that:

$$U_{ijs} = x'_{ijt}\beta_i + e_{ijt} \quad (1)$$

where $x'_{ijt}\beta_i$ is referred to as the systematic utility component and e_{ijt} is the error term assumed to be extreme value (Gumbel) distributed, independent of x'_{ijt} and uncorrelated across individuals or choices.

LCM Model Specification

LCMs enable estimation of the utility function taking account of respondent heterogeneity. The model specification assumes a fixed (finite) number of classes that respondents are statistically allocated into. The LCM, we employed, modifies equation (1) with the inclusion of an additional subscript, s, that specifies the classes:

$$U_{ijt|s} = x'_{ijt}\beta_i + \varepsilon_{ijt|s} \quad (2)$$

It can then be shown that the probability that respondent *i*, belonging to class *s*, will choose an alternative *j* from a choice set of *t* is:

$$Pr_{ijt|s} = \left(\frac{e^{x'_{ijt}\beta_s}}{\sum_{j=1}^{J} e^{x'_{ijt}\beta_s}} \right) \quad (3)$$

As is standard, we employ a MNL to assign respondents *i* to a specific class *s*:

$$Pr_{is} = \left(\frac{e^{z_i\alpha'_s}}{\sum_{s=1}^{S} e^{z_i\alpha'_s}} \right) \quad (4)$$

where α_s is a vector of parameters to be estimated for each s and z_i is a vector of socio-economic variables explaining class membership. It follows that conditional on a specific class membership that the probability that a *i* selects *j* from choice set of *t* is:

$$Pr_{ij|s} = \prod_{t=1}^{T} Pr_{ijt|s} \quad (5)$$

We estimate the LCM by combining equations (4) and (5) as follows:

$$Pr_{ij} = \sum_{s=1}^{S} \left(\frac{e^{z_i\alpha'_s}}{\sum_{s=1}^{S} e^{z_i\alpha'_s}} \prod_{t=1}^{T} \left(\frac{e^{x'_{ijt}\beta_s}}{\sum_{j=1}^{J} e^{x'_{ijt}\beta_s}} \right) \right) \quad (6)$$

We estimate equation (6) by selecting the number of classes, *s*, and then using maximum likelihood estimation. Model selection in terms of number of classes is conducted using the model log likelihood, the Akaike Information Criterion (AIC), and the Bayesian Information Criterion (BIC).

Mixed Logit (MXL) Model Specification

In the MXL specification (Train, 2009), β_i is assumed to be a (k×1) vector individual preferences which are independently and identically normal distributed:

$$\beta_i = \beta + \Gamma \nu_i \quad (7)$$

where β_i has a mean β and Γ is a diagonal matrix including the standard deviation of the random parameter distributions of β_i and ν_i is the unobserved random disturbances.

For our preferred model specification, we have assumed that all parameters follow a normal distribution. To estimate the model in Willingness to Pay space, we follow Balcombe et al. (2010). Our utility function is expressed as follows:

$$U_{ijt} = \beta_1 [Cost_{ijt} + \beta_{2,i} Global\ Journal\ Scope_{ij} + \beta_{3,i} Regional\ Journal\ Scope_{ijt} + \beta_{4,i} Access\ Model_{ijt} + \beta_{5,i} Impact\ Factor_{ijt} + \beta_{6,i} No\ Editorial\ Support_{ijt} + \beta_7 Paid\ Editorial\ Support_{ijt} + \beta_8 Peer\ Review_{ijt} + \beta_9 Society_{ijt}] + e_{ijt} \quad (8)$$

where $\beta_{2,i}$ to $\beta_{9,i}$ are WTP parameters for each individual for the set of attributes. This econometric specification does not have a closed form log-likelihood function meaning estimation is implemented using simulated maximum likelihood. Estimation was undertaken using the software NLOGIT Version 6 (Greene, 2016) employing 1000 Halton draws.

To examine the relationship between our WTP estimates and socio-economic data, we follow Xuan et al. (2021) and Blackhall-Miles et al. (2023) and regress individual attribute WTP estimates against the socio-economic data using panel model specification.

References

Balcombe, K.G., Fraser, I.M. and Di Falco, S. (2010). Traffic Lights and Food Choice: A Choice Experiment Examining the Relationship Between Food Labels and Price. *Food Policy*. 35(3): 211-220.

Blackhall-Miles, R.J., Fraser, I.M. and Roberts, D.L. (2023). Investigating consumer preferences for known wild provenance of plants within the horticultural trade. *Plants, People, Planet*, *5*(3), pp.398-407.

Greene, W.H. (2016). NLOGIT 6 reference guide. Econometric Software, Plainview, New York. https://www.limdep.com/features/whatsnew_nlogit.php

Train, K. E. (2009). Discrete choice methods with simulation. Cambridge University Press.

Xuan, B.B., Armstrong, C.W., Ankamah-Yeboah, I., Hynes, S. and Needham, K. (2021), Valuing high-seas ecosystem conservation. *Conservation Biology*, 35: 1405-1416.

Appendix 6: Summary of statistical measures of model fit for Multinomial logit (MNL), Latent Class Models (LCM) and Correlated Mixed Logit in Willingness to Pay Space (MXL).

**Model**	**k**[a]	**LL**[b]	**AIC**[c]	**AIC/N**	**BIC**[d]	**R^2**	**S1**[e]	**S2**	**S3**	**S4**
MNL	10	-14932.3	29884.6	2.448	29958.8					
LCM2	24	-13229.5	26506.9	2.171	26685.1	0.218	0.449	0.55		
LCM3	38	-12766.6	25609.3	2.097	25891.3	0.245	0.234	0.309	0.457	
LCM4	52	-12471.6	25047.2	2.051	25433.2	0.262	0.224	0.374	0.271	0.131
MXL	48	-11982.7	24061.3	1.971	24417.1	0.292				

[a]Number of parameters.
[b]Log Likelihood.
[c]Akaike's information criterion.
[d]Bayesian information criterion.
[e]The proportion of respondents by LCM segment (S)

Appendix 7: Model Results for the Correlated Mixed Logit in Willingness to Pay Space (MXL). All random parameters Normally Distributed. All coefficients except ASC are Willingness to Pay Estimates. Significance levels: *P < 0.1, **P < 0.05, *** P<0.01. ASC – Alternative specific constant.

**Attributes**	**Coefficients**		**SE**[a]	**SD**[b]		**SE**
ASC[c]	-1.266	***	0.077			
Global Journal Scope	75.339		209.321	3947.86	***	178.43
Regional Journal Scope	338.007	***	99.289	1536.99	***	113.69
Access Model	252.100	**	108.186	1958.84	***	120.28
Impact Factor	13.736	**	5.427	111.31	***	5.37
No editorial support	-1313.09	***	131.963	2293.11	***	185.14
Paid editorial support	-1910.68	***	160.448	2555.07	***	212.87
Peer Review Model	795.326	***	95.985	485.82	***	139.45
Society	130.394		89.459	481.12	***	173.97

Notes:
a - SE = Standard error
b – SD = Standard deviation
c – ASC is a fixed parameter

Appendix 8: Socio-Economic Determinants of Willingness to Pay by attribute using Pooled OLS Fixed and Random Effects Panel Models. Significance levels: **P* < 0.1, ***P* < 0.05, *** *P*<0.01. Excluded level is Regional Journal Scope. GLS Random Effects statistically significant as able to reject null for Bresuch-Pagan and unable to reject null for the Hausmann test.

	**Pooled OLS**		**Fixed Effect**		**GLS Random Effects**	
**Variables**	**Coeff**	**SE[a]**	**Coeff**	**SE[a]**	**Coeff**	**SE[a]**
Constant	500.04***	75.73	22.54	69.49	14.59	73.89
NoPaper	−3.55	2.54	−5.25*	2.77	−4.69*	2.81
Age	−9.44***	1.63	−9.46**	1.74	−9.46**	1.68
HighInc	273.16***	39.60	239.99***	42.72	252.29***	41.57
**Dummies**						
Global	−379.99***	107.27				
Access	−96.33	63.43				
IF	−246.83***	39.78				
NoEd	−1524.52***	71.88				
PaidEd	−2170.39***	74.35				
Peer	535.47***	40.70				
Society	−127.32***	41.33				
R^2	0.216		0.32			
F Test (Model)	582.47***		20.07***			
F Test (Group Dummies)			8.57***			
Regression χ^2 (3)					65.34***	
Breusch-Pagan χ^2 (1)					1356.25***	
Hausman χ^2 (3)					2.64	
N	8200		8200		8200	

Notes: a - SE = Standard error

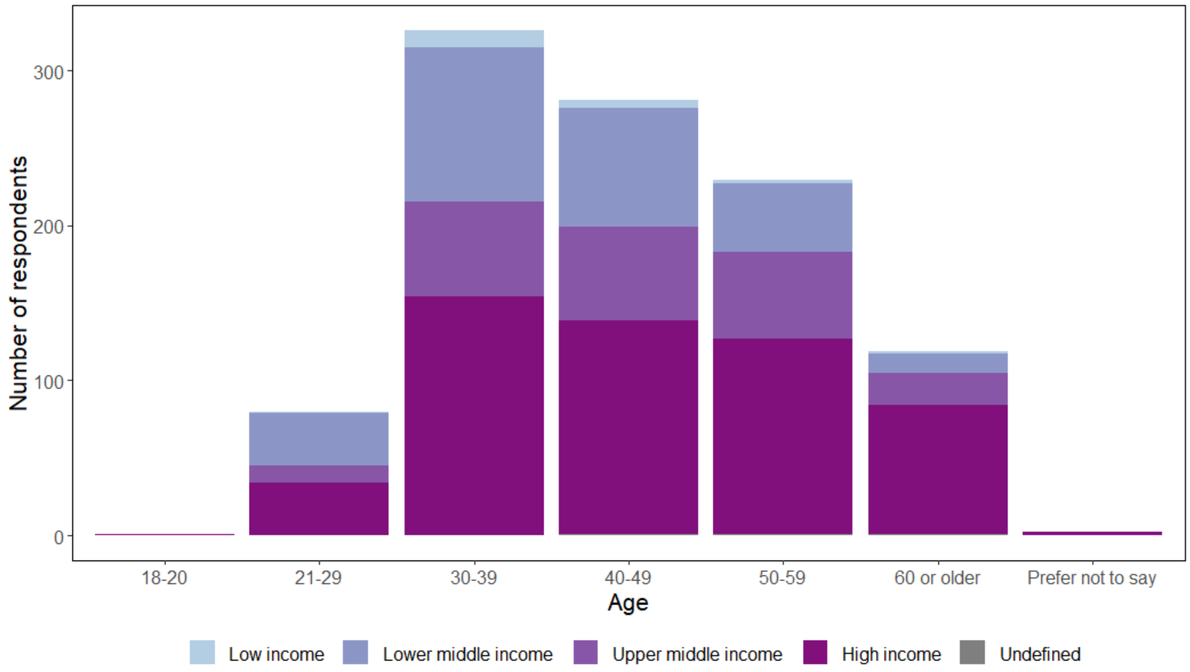

Appendix 9. Summary of respondent demographics between age classes and nationality by income group as defined by the World Bank (2022).

Appendix 10: Article processing charges (excl. taxes), publishing options, and publisher for each journal included in our study. Article processing charges represent fees for full-length, original research articles in $USD for May 2024.

Conservation-related journals	Publishing option	Article processing charge ($)	Publisher
Aquatic Conservation: Marine & Freshwater Ecosystems	Hybrid	4,600	John Wiley and Sons Ltd
Biodiversity and Conservation	Hybrid	3,790	Springer Nature
Biological Conservation	Hybrid	3,570	Elsevier
Conservation Biology	Hybrid	Society members 2,592; Non-members 3,240	John Wiley and Sons Ltd
Conservation Letters	Gold open access only	Society members 2,584; Non-members 3,230	John Wiley and Sons Ltd
Conservation Physiology	Gold open access only	Society members 2,195*; Non-members 2,439*	Oxford University Press
Conservation Science and Practice	Gold open access only	Society members 1,632; Non-members 2,040	John Wiley and Sons Ltd
Diversity and Distributions	Gold open access only	Society members 3,120; Non-members 2,496	John Wiley and Sons Ltd
Ecological Engineering	Hybrid	3,650	Elsevier
Environmental Conservation	Hybrid	3,450	Cambridge University Press
Global Ecology and Conservation	Gold open access only	2,230	Elsevier
Human-Wildlife Interactions	Gold open access only	2,125	Frontiers
Journal for Nature Conservation	Hybrid	2,560	Elsevier
Journal of Applied Ecology	Hybrid	Society members 2,625; Non-members 3,500	John Wiley and Sons Ltd
Landscape Ecology	Gold open access only	4,390	Springer Nature
Nature Conservation	Hybrid	2,560	Elsevier
Oryx	Gold open access only	Member rate 2,933; Non-members 3,450	Cambridge University Press
Wildlife Society Bulletin	Gold open access only	Society members 1,980; Non-members 2,200	John Wiley and Sons Ltd

*Exchange rate from GBP to USD 1.2710 (May 21st 2024).
